# Somatosensory inputs by application of KinesioTaping: effects on spasticity, balance, and gait in chronic spinal cord injury

**DOI:** 10.3389/fnhum.2014.00367

**Published:** 2014-05-30

**Authors:** Federica Tamburella, Giorgio Scivoletto, Marco Molinari

**Affiliations:** Spinal Cord Unit, Clinical Movement Analysis and Research Laboratory, IRCCS Santa Lucia FoundationRome, Italy

**Keywords:** spinal cord injury, KinesioTaping, balance, gait, Electromyography

## Abstract

**Introduction:** Leg paralysis, spasticity, reduced interlimb coordination, and impaired balance are the chief limitations to overground ambulation in subjects with incomplete spinal cord injury (SCI). In recent years, the application of KinesioTaping (KT) has been proposed to enhance sensory inputs, decreasing spasticity by proprioception feedback and relieving abnormal muscle tension. Because no studies have examined KT-based techniques in SCI subjects, our goal was to analyze the effects of ankle joint KT on spasticity, balance, and gait.

**Materials and Methods:** A randomized crossover case control design was used to compare the effects of KT and conventional nonelastic silk tape (ST) in 11 chronic SCI subjects, AIS level D, with soleus/gastrocnemius (S/G) muscle spasticity and balance and gait impairments. Treatment: 48 h of treatment with KT or ST was followed by 48 h with the other technique after 1 week. A single Y-strip of Cure^©^ tape (KT) and ST was to the S and G muscles with 0% stretch. Before and 48 h after of application of KT and ST, clinical data on the range of motion (ROM), spasticity, clonus, pain, balance, and gait were collected. Stabilometric platform assessment of center of pressure (COP) movements; bidimensional gait analysis; and recording of electromyographic (EMG) activity of the S, G, and tibialis anterior and extensor hallucis lungus muscles were also performed.

**Results:** Only KT had significant effects on spasticity (*p* < 0.05), clonus (*p* < 0.001) and COP movements (*p* < 0.05), kinematic gait parameters (*p* < 0.001), and EMG activity (*p* < 0.001). Comparison between ST and KT improvements pointed out significant differences as concerns ROM (*p* < 0.001), spasticity (*p* < 0.001), clonus (*p* < 0.001), pain (*p* < 0.001), COP parameters (*p* < 0.05), and most kinematic gait data (*p* < 0.05).

**Discussion:** Short-term application of KT reduces spasticity and pain and improves balance and gait in chronic SCI subjects. Although these data are promising, they require confirmation in a larger cohort of patients.

## Introduction

In designing effective gait rehabilitation programs after spinal cord injury (SCI), knowledge of the neuronal mechanisms that mediate and, in particular, influence the afferent feedbacks in the function of the damaged spinal cord is paramount (Hubli and Dietz, 2013). Locomotion requires continuous modulation of spinal central pattern generator (CPG) circuits to adapt to the everchanging environment. Feedback from a variety of sources, such as visual, vestibular, somatosensory, and proprioceptive circuits, must be interpreted and integrated into CPG activity to generate locomotion that is effective under all conditions (Hubli and Dietz, 2013). In this complex framework, sensory feedback and context-specific gait requirements interact in affecting muscle synergies (Horak and Nashner, 1986).

In recent years, increasing cutaneous stimuli through neuromuscular KinesioTaping (KT) has been proposed to enhance somatosensory inputs (Halseth, 2004). Alexander et al. reported decreased H-reflex amplitude after KT of the trapezius, suggesting that it influences muscle tone (Alexander et al., 2003). This KT-dependent H-reflex decline indicates that it is inhibitory and adjusts muscle activity through proprioception feedback (Lin et al., 2011). KT has been used in neurological pathologies (Kilbreath et al., 2006; Karadag-Saygi et al., 2010; Cortesi et al., 2011), including stroke and multiple sclerosis, and various orthopedic disorders (Alexander et al., 2003; Halseth, 2004; Thelen et al., 2008; Lin et al., 2011), generally improving muscle tone, range of motion, center of pressure balance parameters, and pain symptoms.

No study has addressed the use of KT in subjects with SCI. Major gait impairments in incomplete SCI are caused by ankle spasticity (Scivoletto et al., 2008; Arazpour et al., 2013) and decreased balance (Scivoletto et al., 2008; Tamburella et al., 2013a), both of which are positively affected by KT in neurological (Cortesi et al., 2011) and nonneurological disorders (Alexander et al., 2003; Halseth, 2004; Lin et al., 2011). Thus, we examined KT treatment in controlling ankle muscle tone in subjects with incomplete SCI, determining its effects on spasticity, balance, and gait by clinical and instrument-based evaluations.

## Materials and methods

### Study design—population

A randomized crossover case control design was used to compare the effects of KT and conventional nonelastic silk tape (ST) on ankle muscles in subjects with chronic incomplete SCI. Patient selection was based on the clinical assessment, per the American Spinal Injury Association (ASIA) standards for neurological status, and on the degree of ankle spasticity, per the modified Ashworth scale (MAS). The inclusion criteria were chronic SCI lesion (i. e., at least 12 months post-injury), AIS level D, and MAS higher than 2 bilaterally in the soleus/gastrocnemius muscles. The exclusion criteria were the presence of other neurological or orthopedic impairments, participation in other studies, and pharmacological treatment for spasticity in the previous 4 weeks. This study was approved by the local ethics committee.

From January 1, 2013 to April 30, 2013, 33 consecutive patients who were admitted to the Spinal Cord Rehabilitation outpatient service of Santa Lucia Foundation were examined by an experience neurologist (Giorgio Scivoletto), of whom 11 subjects met the inclusion criteria. The demographics and clinical features of the SCI subjects are reported in Table 1.

**Table 1:** **Patients' clinical and epidemiological data**.

**Patients**	**Sex**	**Age**	**Weight(Kg)**	**Height (cm)**	**Etiology**	**Lesion level**	**Years since SCI**	**MAS**	**WISCI level**
PT1	M	34	85	1.82	Traumatic	C6	4	3	13
PT2	M	69	75	1.65	Traumatic	C6	8	2	18
PT3	F	35	60	1.76	Nontraumatic (Degenerative)	T9	4	5	19
PT4	M	51	74	1.73	Nontraumatic (Vascular)	C6	3	2	18
PT5	F	41	60	1.64	Nontraumatic (Vascular)	T6	4	2	19
PT6	M	52	80	1.78	Traumatic	C6	3	2	20
PT7	F	77	67	1.66	Nontraumatic (Vascular)	T10	7	2	19
PT8	M	58	66	1.73	Nontraumatic (Tumoral)	T11	2	4	19
PT9	F	41	55	1.7	Nontraumatic (Tumoral)	T7	10	4	20
PT10	M	72	81	1.64	Nontraumatic (Degenerative)	C7	6	3	13
PT11	F	38	64	1.6	Traumatic	T8	12	3	20
Mean		52	70	170			5.72	2.9	18
*SD*		16	10	0.07			3.19	1.04	2.57

### Intervention: KT and ST treatment

**Figure 1:**
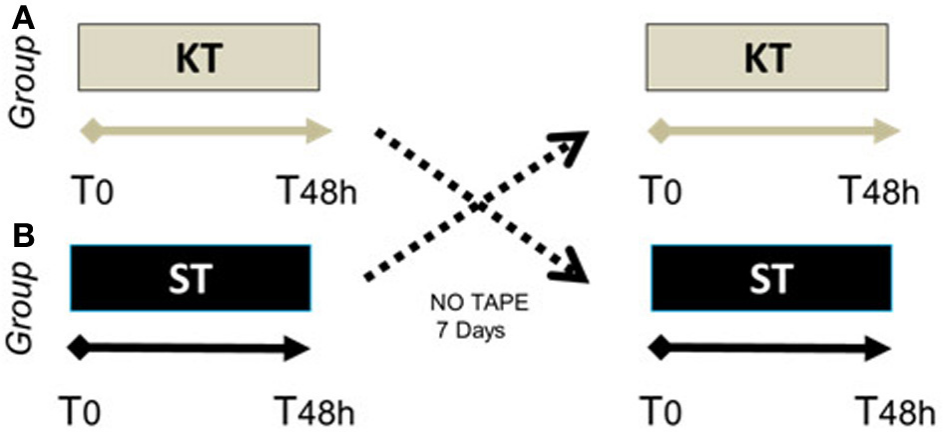
**Randomized crossover case control study schema**. Group **(A)** (*n* = 6), SCI patients who underwent 48 h of KT, followed by 48 h of ST 1 week later. Group **(B)** (*n* = 5), SCI patients who received 48 h of ST treatment, followed by 48 h of KT 1 week later.

After enrollment, SCI subjects were randomized into 2 treatment groups. Group A (*n* = 6) underwent 48 h of KT, followed by 48 h of ST 1 week later. Group B (*n* = 5) received 48 h of ST treatment, followed by 48 h of KT treatment after 1 week (Figure 1). All subjects underwent clinical and instrumental evaluations before (T0) and immediately after treatment (T_48 h_). Electromyography (EMG) was performed only in Group B before, during, and after KT treatment. A certified KT practitioner (Federica Tamburella) administered all taping procedures. Clinical and instrumental outcomes were measured at T0 and T_48 h_ after removal of the KT by a different researcher (L.M.) who was blinded to the treatment.

**Figure 2:**
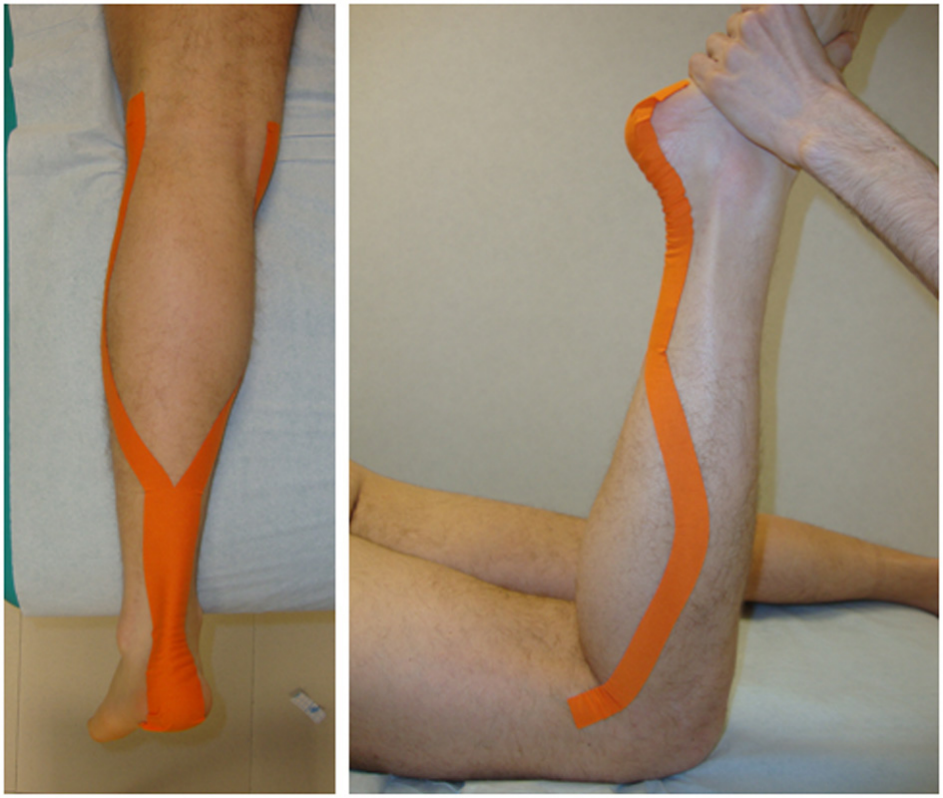
**Application of KT and ST tape to soleus and gastrocnemius ankle muscles**.

KT and ST were applied bilaterally to the plantar-flexor ankle muscles, soleus (S), and gastrocnemius (G), per Luque-Suarez et al. (Luque-Suarez et al., 2014). Standard 5-cm single-strip nonelastic silk tape and Cure^©^ tape were used for the ST and KT, respectively. Y-strip tapes were applied to the S and G muscles with the subject in a prone position, the with knee extended and the ankle in 90° passive dorsiflexion. Both tapes were applied directly to the skin using a decompressive muscle technique, with 0% stretch, from the calcaneus to the medial and lateral femoral condyles (Figure 2). To maximize their adhesion, the tape strips were warmed by rubbing them in the hands several times on the application zone (Luque-Suarez et al., 2014).

### Setup and evaluation of outcomes

All assessments were performed by the same examiner at the same time each day before application of the tape (T_0_) and after 48 h (T_48 h_) of KT or ST treatment. Neurological status was assessed using the American Spinal Injury Association (ASIA) and ASIA Impairment Scale (AIS) (American Spinal Injury Association, 2000). AIS levels A and B indicate complete motor lesions, and AIS levels C and D reflect incomplete motor lesions. Active and passive range of motion (ROM) was measured using a standard manual goniometer (Fong et al., 2011).

The Modified Ashworth Scale (MAS) (Gregson et al., 1999) was used to evaluate ankle spasticity. Spasms, clonus, and pain were scored using the Penn modified Spasm Frequency Scale (PSFS) (Penn, 1988), Spinal Cord Assessment Tool for Spastic Reflexes subscale for clonus assessment (SCATS) (Benz et al., 2005), and Global Pain Scale (GPS) (Wewers and Lowe, 1990), respectively. Balance and gait were assessed using the Berg Balance Scale (BBS) (Lemay and Nadeau, 2010), Walking Index for Spinal Cord Injury (WISCI) (Ditunno and Dittuno, 2001), 10-meter walk test (10WT) (Rossier and Wade, 2001), 6-min walking test (6MWT) (Poole-Wilson, 2000), and timed up and go test (TUG) (Podsiadlo and Richardson, 1991). Walking time tests were performed using a self-selected walking device, if needed (Patrick et al., 2011) and scored using the WISCI, as reported in Table 1. All subjects we subjected to instrument-based balance and gait analyses as detailed below.

The visual analog scale (VAS) was administered at T_48 h_ to assess perception of reductions in spasticity. Patients were asked to quantify the reduction in spasticity due to the tape, on a scale from 0 (no reduction in spasticity) to 10 (maximum reduction in spasticity). Electromyography (EMG) analyses were performed only for KT-treated subjects in Group B.

### Evaluation of balance

Stabilometric parameters were analyzed using a 320 × 75-cm (length × width) static force platform (Platform BPM 120, Physical Support Italia, Italy). The signals were amplified and acquired using dedicated software (Physical Gait Software Vv. 2.66, Physical Support Italia, Italy). Static stability was assessed per Tamburella et al. for chronic SCI subjects (Tamburella et al., 2013a). Patients stood barefoot in a natural and relaxed position with their arms by their sides, without shoes and with both heels lined up, under 2 sensory conditions: eyes open (OE) facing forward to a target 1.5 m away and eyes closed (CE). For each condition, the recording time was set to 51.2 s. and measurements were recorded 3 times and averaged.

We considered the following quantitative COP parameters:

- Length indicators: path length (L, mm), mean (V, L divided by trial duration), anteroposterior velocity (V_AP_, mm/s) laterolateral velocity (V_LL_, mm/s), and mean position of planar laterolateral COP.
- Surface indicators: area of the ellipse encompassing 90% of COP samples (A, cm^2^) and length of its semiaxes (X and Y, cm).

### Evaluation of gait

Locomotion kinematic gait data were recorded and analyzed using the bidimensional KineView Motion System ® (Kineview, Hafnarfjordur, Iceland) per the protocol for chronic SCI subjects in Tamburella et al. (2013a,b), based on 3 strides at a self-determined velocity. Spatial movements of the lower extremity segments were monitored, based on the position of passive markers that were placed per the Helen Hayes biomechanical model (Kadaba et al., 1990). Kinematic data were reconstructed offline using Matlab (Mathworks, Inc., version 7.1, Natick, Massachusetts, USA).

The following kinematic data were considered: speed (m/s), cadence (N° step/min), stride length (STRIDE: mean of right and left stride in m), stance phase (STANCE: mean of right and left stance phase expressed as the percentage of gait cycle), and double-time support phase (DTS: mean of right and left double-time support phase, expressed as the percentage of gait cycle). STRIDE was defined as the event between 2 successive instances of foot-ground contact. STANCE was defined as the event from foot-ground contact to liftoff, and DTS was the time during which both feet were in contact with the ground (Huxham et al., 2006; Tamburella et al., 2013a,b). Foot-ground contact was determined manually from video recordings (Tamburella et al., 2013a,b). All gait variables were averaged from the kinematic data of the 3 trials.

### EMG assessment

For Group B patients, surface EMGs of tibialis anterior (TA), extensor hallucis longus (EHL), S, and G muscle activity were analyzed. Recordings were made before (T_0_), 5 minutes after KT was applied (T_1_), and after the KT was removed (T_48 h_). EMG data were acquired through 4 wireless EMG sensors, 1 for each muscle, affixed per SENIAM recommendations (Oliveira et al., 2012) using EMG Delsys. EMG data were processed using EMG Works Analysis (Delsys, Boston, USA) using a pass-band filter between 10 and 450 Hz, and successively a 50-Hz notch filter. Root mean square (RMS) values, with a window of 0.250 and an overlap of 0.0625, were obtained from the filtered data. Data on each muscle were then imported into Matlab (Mathworks, Inc., version 7.1, Natick, Massachusetts, USA) to analyze muscle coactivation by calculating the coactivation index (CI) (Kellis et al., 2003).

$$CI=\frac{\int EMG(S+G)}{\int EMG[(TA+EHL)+(S+G)]}\cdot 100$$

CI is a relative measure of antagonist (S and G) contribution to total activation (S and G + TA and EHL) during the dorsiflexion task (Kellis et al., 2003). Thus, an increase in CI reflects a rise in co-contraction. CI ranged from 0 to 100%, with 100% indicating full muscle coactivation, defined as coactivation (i. e., simultaneous activity) of all ankle muscles. EMG data were recorded while patients were asked to perform maximal voluntary contraction (MVC) during 5 dorsiflexion active movements lying down with knees flexed and extended. Data were averaged across the 5 active tasks.

### Statistical analysis

No participant withdrew from the trial, and all outcome measures were obtained for all SCI subjects. Descriptive statistics were generated for all variables. Prior to the statistical comparisons, normal distribution of the data was confirmed by Kolmogorov-Smirnov test.

Treatment effects were analyzed by grouping the KT and ST data on Group A and B subjects. Paired *t*-test was used to compare the effects of treatment, evaluated as T0 vs. T_48 h_, for each KT or SK treatment groups. At T0 and T_48 h_, KT and ST were compared by independent *t*-test and Mann-Whitney *U*-test for ordinal and nonordinal variables, respectively.

For each clinical and instrument-based parameter, the percentage of improvement due to KT and ST was calculated as follows:

Percentage of improvement = [(T_48 h_ data-T_0_ data)/T_0_]^*^100. Treatment effects on percentage of improvement data were analyzed by independent *t*-test or Mann-Whitney *U*-test when appropriate.

CI data on KT-treated Group B patients were analyzed by repeated measures ANOVA, with time (T0 vs. T_1_ vs. T_48 h_) as the main within-group factor, followed by Bonferroni *post-hoc* test when the ANOVA results reached significance.

Statistical significance was considered at *p* < 0.05 (^*^p < 0.05, ^**^p < 0.005, ^***^p < 0.001). All statistical tests were performed using the Statistical Package for the Social Sciences Software (SPSS), version 12.0 (Chicago, IL).

## Results

No clinical or instrument-based assessment differed significantly between Groups A and B at T_0_ (Table 2) (*p* > 0.05).

**Table 2:**
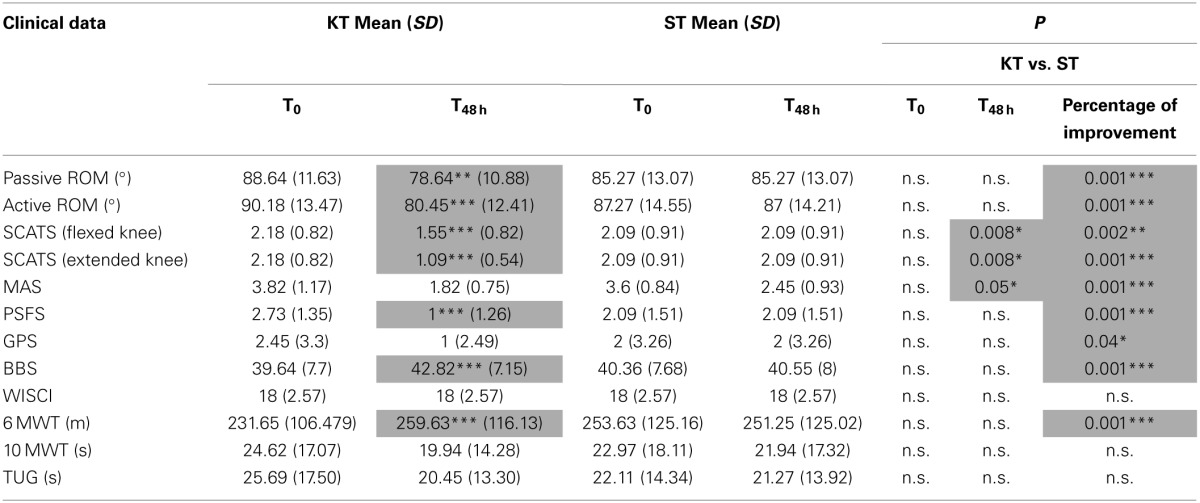
**Clinical and instrumental assessment**.

The KT, T_ıt 48 h_ column lists the statistical comparison of T0 vs. T_ıt 48 h_ data. Comparisons between KT and SHAM data at T0 and T_ıt 48 h_ and percentage of improvement between KT and ST groups are reported in the last columns of the table (^*^p < 0.05, ^**^p < 0.005, ^***^p < 0.001). Gray cells indicate significant p-values. n.s., not significant; for clinical scales abbreviations, see main text.

### Clinical assessment

The clinical assessment results are shown in Table 2. As expected, almost no changes were observed between T_0_ and T_48 h_ in the ST group. Conversely, vs. T_0_, KT treatment at T_48 h_ significantly improved passive (*p* < 0.005) and active ROM (*p* < 0.001), SCATS score with the knees flexed and extended (*p* < 0.001), PSFS (*p* < 0.001), BBS (*p* < 0.001), and 6MWT (*p* < 0.001). Compared with ST, KT had significant treatment effects T_48 h_ on SCATS with the knees flexed and extended (*p* < 0.05) and on MAS (*p* < 0.05).

Based on percentage of improvement values, we noted significant treatment effects on active and passive dorsiflexion ROM (*p* < 0.001), pathological reflex with the knees flexed (*p* < 0.005) and extended (*p* < 0.001), PSFS (*p* < 0.001), GPN (*p* < 0.001), BBS, and 6MWT (*p* < 0.001).

With regard to perception of spasticity, VAS score was 7.9 ± 1.2 after KT and 2.5 ± 1.3 after ST (*p* < 0.05).

### Evaluation of balance

Between T0 and T_48 h_, KT significantly improved L, C, V_LL_, and V_AP_ under the OE and CE conditions (*p* < 0.05). Compared with ST, at T_48 h_, KT had significant treatment effects on L, V, V_LL_ (*p* < 0.05), and V_AP_ (*p* < 0.001) under the OE condition and on L and V under the CE condition (*p* < 0.05). Based on percentage of improvement values, significantly treatment effects were pointed out for L, V, and V_AP_ (*p* < 0.05) under the OE and CE conditions and enhanced A, Y, and V_LL_ under the CE condition (A and V_LL_: *p* < 0.05; Y and V_AP_ *p* < 0.005) (Table 3).

**Table 3:**
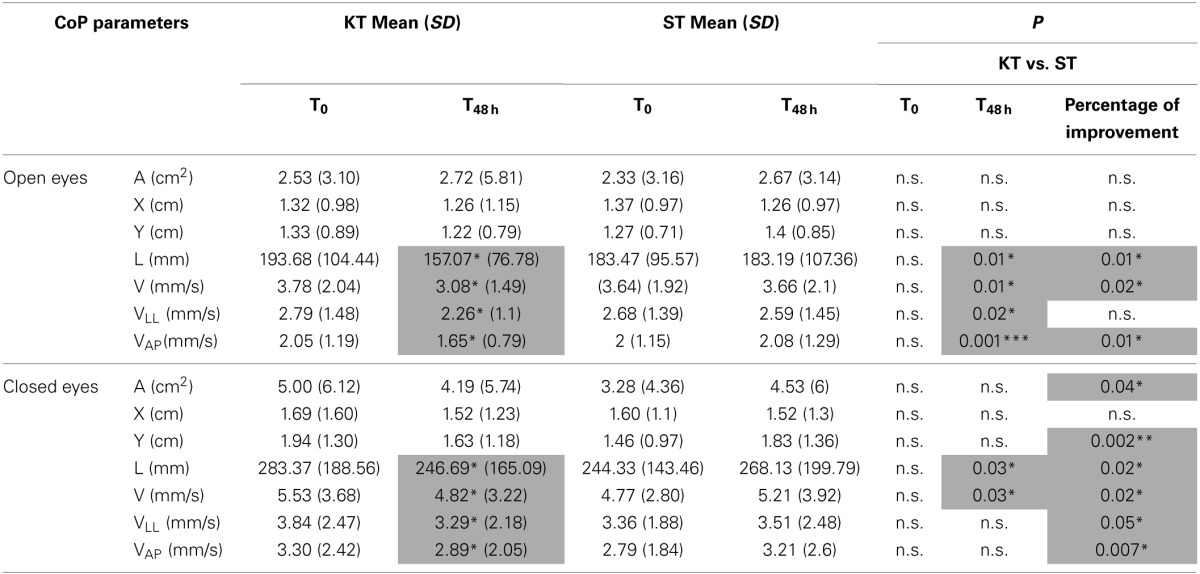
**Balance assessment**.

The KT, T_ıt 48 h_ column lists the statistical comparison of T0 vs. T_ıt 48 h_ data. Comparisons between KT and SHAM data at T0 and T_ıt 48 h_ and percentage of improvement between KT and ST groups are reported in the last columns of the table (^*^p < 0.05, ^**^p < 0.005, ^***^p < 0.001). Gray cells indicate significant p-values. n.s., not significant; for abbreviation of COP parameters, see main text.

### Evaluation of gait

The effects of KT on STRIDE, STANCE, and DTS at T_48 h_ vs. T0 were significant (*p* < 0.001). Further STRIDE (*p* < 0.001), STANCE, and DTS (*p* < 0.005) improved with KT at T_48 h_ compared with ST. Comparison of percentage improvement values demonstrated significant treatment effects for all kinematic parameters (speed, cadence, and DTS: *p* < 0.05; STRIDE and STANCE: *p* < 0.001) (Table 4).

**Table 4:**
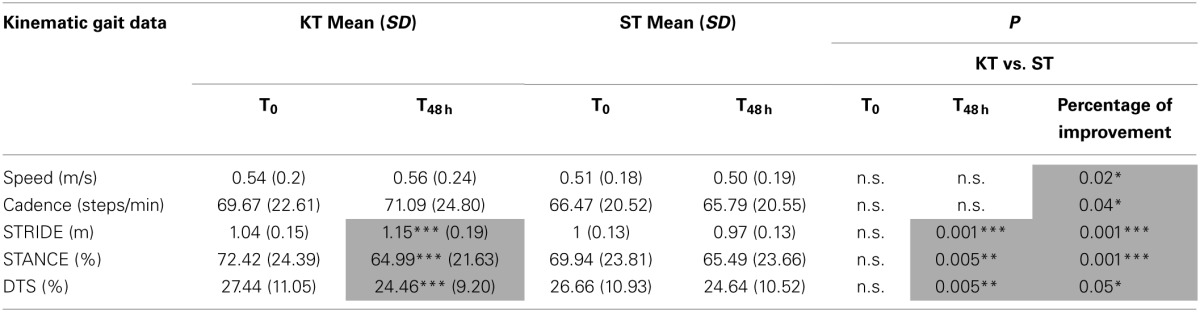
**Gait assessment**.

The KT, T_ıt 48 h_ column lists the statistical comparison of T0 vs. T_ıt 48 h_ data. Comparisons between KT and SHAM data at T0 and T_ıt 48 h_ and percentage of improvement between KT and ST groups are reported in the last columns of the table (^*^p < 0.05, ^**^p < 0.005, ^***^p < 0.001). Gray cells indicate significant p-values. n.s., not significant; DTS, double-time support phase.

### Assessment of EMG CI

**Figure 3:**
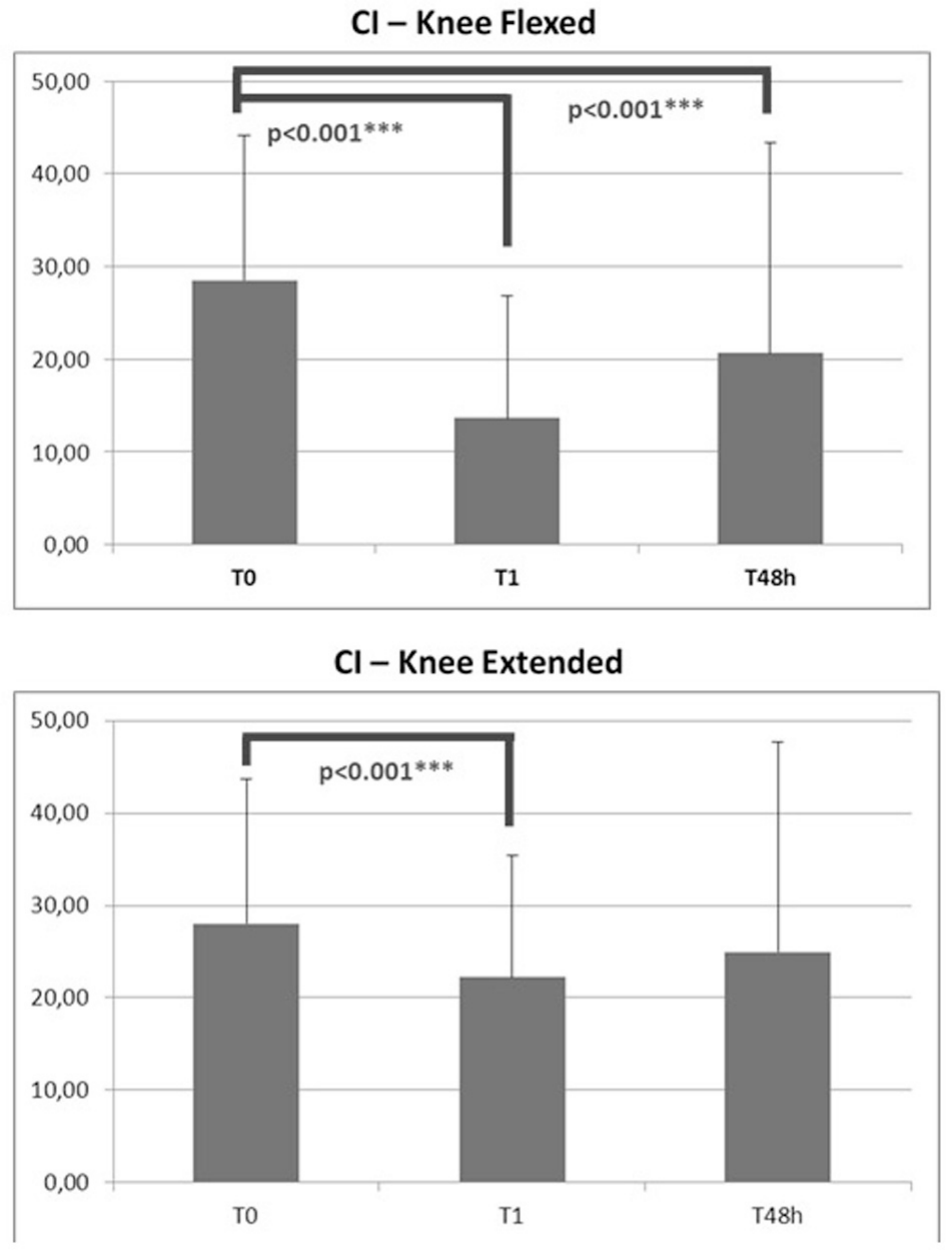
**CI index at T_0_, T_1_, and T_48 h_ for EXP group**. ^***^p < 0.001.

CI, as assessed with the knees flexed or extended, declined significantly immediately after application of KT (*p* < 0.001 − F [19.046]). After 48 h of treatment, this effect was maintained only with the knees flexed (*p* < 0.001 − F [0.820]) (Figure 3).

To determine the most notable effects of KT, the results were divided into primary and secondary outcome measures, as reported in Table 5. MAS, BBS, CoP V, 6MWT, STRIDE, and STANCE were identified as the most important outcomes, and the remaining data were considered secondary outcomes and used to evaluate additional effects of the intervention.

**Table 5:**
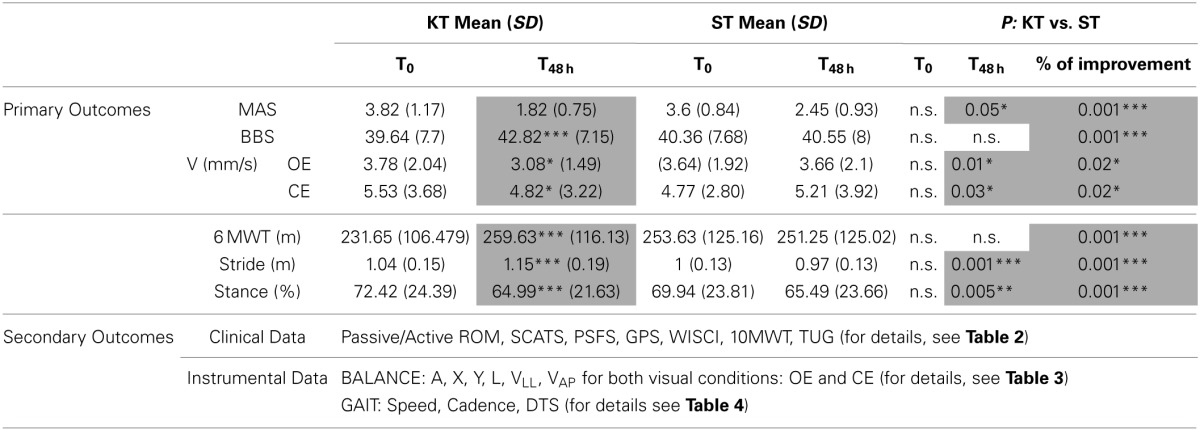
**Primary and secondary outcome measures**.

Clinical and instrumental data on spasticity, balance, and gait have been divided into primary and secondary outcome measures. The KT, T_ıt 48 h_ column lists the statistical comparison of T0 vs. T_ıt 48 h_ data. Comparisons between KT and SHAM data at T0 and T_ıt 48 h_ and percentage of improvement between KT and ST groups are reported in the last columns of the table (^*^p < 0.05, ^**^p < 0.005, ^***^p < 0.001). Gray cells indicate significant p-values. n.s., not significant; for abbreviations, see main text.

## Discussion

**Figure 4:**
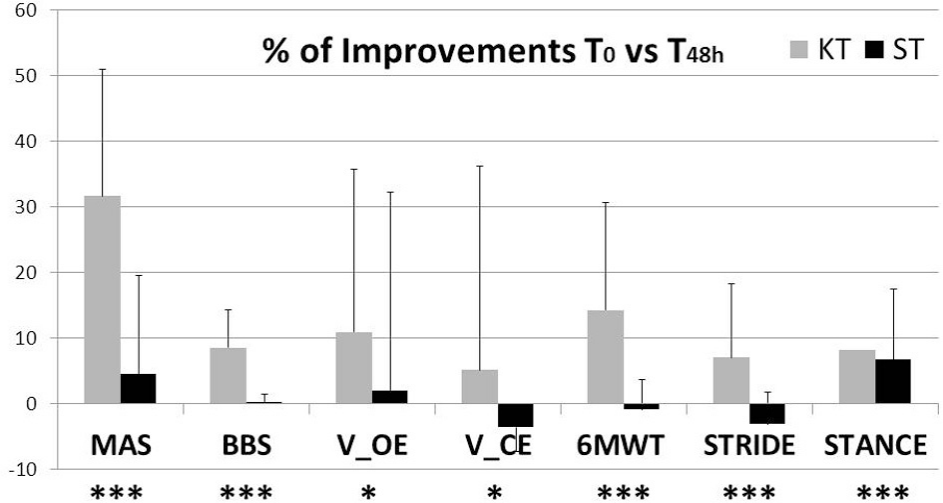
**Percentage of improvement due to 48-h application of KT or ST**. ^*^p < 0.05, ^***^p < 0.001.

In this study, we examined the effects of KT treatment in chronic incomplete SCI subjects compared with nonelastic ST on functional relevant aspects of the post-SCI condition—, i. e., ankle muscle spasticity, balance, and gait. By MAS and analysis of functional balance and gait, 48 h of KT treatment improved all primary outcomes: MAS, BBS, V CoP, 6MWT, STRIDE, and STANCE (Figure 4), indicating better functional status after KT, with reduced spasticity and improved balance and gait. In general no adverse events were observed, and subjects reported no discomfort during KT treatment.

KT is used to enhance sensory inputs, decreasing spasticity through proprioception feedback and relieving abnormal muscle tension, in healthy athletic subjects (Thelen et al., 2008; Lin et al., 2011). Few studies have examined KT in neurological lesions, such as multiple sclerosis (Cortesi et al., 2011) and stroke (Kilbreath et al., 2006; Karadag-Saygi et al., 2010). In multiple sclerosis, Cortesi et al. observed positive effects of KT of the ankle on COP balance parameters, suggesting that ankle taping helps stabilize body posture immediately (Cortesi et al., 2011). In stroke patients, KT of the gluteus muscles increases hip extension during gait, suggesting that muscle activation improves through cutaneous stimuli (Kilbreath et al., 2006), whereas no positive effects were obtained by combining ankle KT and botulinum toxin to reduce plantar flexor spasticity (Karadag-Saygi et al., 2010). No data are available on the effects of KT in SCI subjects.

The crossover paradigm that we used allowed us to blind subjects to the treatment allocation and limit the risk of compliance in analyzing the effects of KT vs. ST (Mills et al., 2009). To prevent overflow effects of KT, an interval of 7 days separated application of the tapes (Chen et al., 2013; de Hoyo et al., 2013).

Although significant differences were observed in passive/active ROM, clonus, spasms, BBS, 6MWT, and most COP parameters and kinematic gait data after 48 h of KT, almost no changes were observed after 48 h of ST treatment, as expected, due to the chronic condition. Treatment effects were analyzed by comparing improvements after 48 h of KT and ST treatments. KT reduced MAS and improved active and passive ankle ROM, which was paralleled by a decrease in spasticity-associated symptoms, clonus, and pain. Control of ankle spasticity is paramount in improving balance and gait in SCI subjects (Arazpour et al., 2013). KT had significant therapeutic effects on balance and gait, both of which improved with regard to the clinical scales and stabilometric and kinematic data. After KT treatment, there was better control of balance, as confirmed by the decline in V and L COP parameters (Cortesi et al., 2011; Tamburella et al., 2013a), as well as an improvement in kinematic gait parameters. Decreases in STANCE and DTS reflect improved dynamic postural stability (Tamburella et al., 2013b), which has been suggested to specifically improve gait in subjects with chronic motor incomplete SCI (Tamburella et al., 2013a).

To determine the possible mechanism of these improvements, EMG data were collected in Group B patients before, during, and after KT treatment. CI has been proposed as an index of spasticity in stroke subjects (Hu et al., 2007) and is indicative of fatigue-induced decreases in muscular co-contraction in healthy athletic subjects (Missenard et al., 2008). In this study, we used the CI to evaluate EMG activity of agonist vs. antagonist ankle muscles. A high degree of CI reflects excessive antagonistic muscle contractions during dynamic activities compared with agonist muscle activity, impairing function and increasing the metabolic cost of performing the task (Knarr et al., 2012). Our EMG data demonstrated a significant reduction in CI with KT, suggesting improved motor outcome (Hu et al., 2009) and confirming our clinical data on spasticity.

Notably, CI improved immediately after application of KT—an effect that was maintained, although slightly reduced, after 48 h. The lack of significance of the CI data at 48 h with the knee extended confirm the high variability of spasticity measurements in this posture compared with the knee flexed (Figure 3). The significant reduction in CI due to KT might be explained by 2 reasons: the increase in EMG activation of the TA and EHL and the decreased co-contraction phase of the antagonist S and G muscles. Considering the findings of Alexander et al. (2003), in which amplitude of the H-reflex decreased after KT of the trapezius in healthy subjects, it is conceivable that KT also adjusts muscle activity by inhibiting proprioception feedback (Lin et al., 2011) also in SCI subjects.

To improve outcomes and methods of applying the tape, it is necessary to understand the mechanism that leads to better upright balance and gait. The effects of KT were clinically significant immediately after its application, implying that the changes were not due to long-term learning, as reported in multiple sclerosis (Cortesi et al., 2011). In addition to the secondary effects of spasticity changes, the alterations in the balance control system might be explained by changes in skin receptor inputs due to application of KT (Morasso and Schieppati, 1999). The mechanical effects of applying tape to the skin might increase skin receptor output, stimulating supraspinal centers and thus enhancing kinesthetic and joint position sense (Simoneau et al., 1997; Halseth, 2004) and improving balance.

In analyzing the effects of KT on gait, sensory components cannot be dismissed. Applying pressure to and stretching the skin with KT can stimulate cutaneous mechanoreceptors and enhance signal information of joint movement or joint position (Hsu et al., 2009) (Riemann and Lephart, 2002). The importance of sensory inputs in influencing the activity of gait central pattern generators (CPGs) is highlighted (Grillner, 2006). In SCI patients, changes in CPG circuits are well documented (Grillner, 2006; Roy et al., 2012) and are learning-dependent, primarily through rhythmic peripheral influences that imposed by the exercise—for instance, during robotic gait training (Curt et al., 2008; van Hedel and Dietz, 2010). The importance and effectiveness of sensory input in modulating stepping in SCI has been demonstrated in a wide range of experiments (Edgerton and Roy, 2009); for example, modulation of sensory information influences spinal circuit reorganization to be effective from milliseconds to months (Roy et al., 2012). Thus, sensory modulation through KT might not only influence spasticity but also intervene in longlasting reorganization of spinal gait circuits. In this theoretical framework, the influences of KT on gait merit studies not only in subjects with SCI but in all neurological gait pathologies.

Subjectively, the VAS results indicate a significant reduction in perception of spasticity after KT treatment and that spasticity is negatively associated with quality of life after SCI (Westerkam et al., 2011).

The significance of the sensory effects of KT must also be considered in analyzing its effects on pain. In our study, despite the short-term treatment, GPS declined significantly after KT and but not with ST. Treatment significance was present when comparing KT and ST GPS improvements. Although this study did not aim to evaluate the effects of KT on pain, our results are consistent with data in chronic low back pain patients (Paoloni et al., 2011) and merit dedicated studies, possibly with longer application times of KT.

In conclusion, KT is a valid technique to reduce spasticity and related symptoms in the short term and improve balance and gait in chronic incomplete SCI subjects. Further studies are needed to determine its long-lasting effects.

### Limitations

The sample size of SCI subjects (*n* = 11) was small, which might have limited the statistical relevance of the study. Nevertheless, the statistical differences were large, rendering the statistical error that was caused by sample size negligible. Further, as suggested by Friston (2012), significant results that are based on a small sample indicate a greater treatment effect than equivalent results in a large sample.

A follow-up study with a longer KT application is necessary to confirm these preliminary data, and a theory on the neurophysiological effects of taping would facilitate the generation of experimental hypotheses.

## Author contributions

Federica Tamburella: Substantial contributions to the conception and design of the work; the acquisition, analysis, and interpretation of data; drafting of the manuscript and revising it with regard to intellectual content. Giorgio Scivoletto: Substantial contributions to the conception and design of the work, the interpretation of data, and revision of the work with regard to intellectual content. Marco Molinari: Substantial contributions to revision of the work with regard to intellectual content and final approval of the version to be published.

### Conflict of interest statement

The authors declare that the research was conducted in the absence of any commercial or financial relationships that could be construed as a potential conflict of interest.
